# Editorial: Deformable object manipulation

**DOI:** 10.3389/fnbot.2022.1122745

**Published:** 2023-01-05

**Authors:** Solvi Arnold

**Affiliations:** Autonomous Intelligence and Systems Laboratory, Department of Mechanical Systems Engineering, Shinshu University, Nagano, Japan

**Keywords:** deformable objects, manipulation, simulation, neural networks, planning

There has been increasing interest in the topic of deformable object manipulation (DOM) from the robotics community over the past few years. Not only do we see an increasing number of papers addressing the topic but also dedicated workshops at major conferences, and even a dedicated competition at IROS. A number of factors are driving this trend. One factor is that the prospect of robots playing an active role in caregiving and household support is becoming more and more tangible. Many practical tasks in such settings require the manipulation of deformables in one form or another. In this Research Topic, the work by De Gusseme and Wyffels specifically focuses on folding clothes, while Kawaharazuka et al. and Arnold et al. also design their tasks with household support settings in mind. Within manufacturing, the handling of rigid objects is increasingly automated, but the handling of deformables lags behind. This naturally puts a spotlight on deformables in manufacturing-focused work. The work by Wang and Yamakawa fits in this context, with high potential for application in e.g. industrial wiring tasks.

Meanwhile, advances in AI are providing new tools and conceptualisations for manipulation problems, making the complexities of DOM perhaps look less daunting than they used to. For researchers with an interest in neural approaches, DOM has become a tantalising challenge. Deformables present high-dimensional state spaces with complex dynamics and numerous sources of uncertainty. This complexity and inherently fuzzy character make for an excellent testbed for neural approaches. Among the contributions to this Research Topic, the works by Kawaharazuka et al. and Arnold et al. pursue such approaches, with neural networks providing predictive capacities for a cloth item's shape development during manipulation.

This Research Topic has fielded varied works in terms of problem settings and approaches. However, a few common themes emerge. We discuss these briefly.

## Material variation

All contributed works touch on the issue of variation in material as a complicating factor in DOM. Kawaharazuka et al. pursue an approach that actively aims to identify material properties, using a self-organising material representation implemented as parametric bias. The inferred properties are used to improve the manipulation trajectory. By contrast, De Gusseme and Wyffels explore trajectory robustness across dimensions of variation, including material factors such as cloth thickness and friction. These approaches illustrate two strategies for increasing robustness to material variation, both with their own strengths and limitations. Material properties are hard to infer from static visual information alone, so material inference strategies must rely on some degree of prior experience with the object. Robustness-focused cloth-agnostic strategies avoid dependency on prior experience but almost necessarily trade robustness for some accuracy. It is easy to see how a dynamic combination of these strategies could be effective. This will likely require an epistemic dimension to the object representation (“what do we know about this object?”). To the best of our knowledge, such avenues remain to be explored.

Material variation features in a different capacity in Wang and Yamakawa's work. In keeping with a manufacturing context, material properties are assumed to be known. This knowledge is effectively used to robustly track linear objects under occlusion. So, whereas Kawaharazuka et al. use object dynamics to fill in otherwise unobservable material properties, Wang and Yamakawa could be said to use material properties to fill in unobservable object dynamics.

Arnold et al. focus on high-level planning. Hardware experiments show potential for generalisation from simulation to real cloth, but also reveal characteristic discrepancies due to the material differences between hardware and simulation. Open questions arising here include how to best account for material variation in high-level planning, and to what extent material-specific adjustments can be pushed down to more granular low-level control.

## The changing role of simulation

The work in this Research Topic also highlights the changing role of simulation in DOM. None of the contributions assume full online use of simulation. Simulation primarily features in a data-generation capacity. Kawaharazuka et al. and Arnold et al. employ simulation data for training. De Gusseme and Wyffels primarily study trajectory robustness but also discuss the applicability of their work for constructing databases for manipulation generation. Wang and Yamakawa employ an object model suitably simplified to the occlusion problem, thereby avoiding the cost of full simulation altogether.

While heavy simulation is relegated to offline data generation, we also observe the use of increasingly advanced simulation setups to improve sim-to-real transfer. Previous work on cloth simulation in the context of computer graphics research has primarily focused on natural cloth motion. For application in robotic manipulation, factors such as realistic friction, cloth stiffness, proper shape stabilisation, and the stability of stacked layers of cloth become important issues. In this Research Topic, we observe the use of environments that improve on a number of these points. Arnold et al. employ ARCSim (Narain et al., 2013), while De Gusseme and Wyffels appear to be the first to use C-IPC (Li et al., 2021) in a cloth manipulation setting.

## Unknowns and uncertainties

Contributions to this Research Topic also emphasise some of the epistemic complexities of DOM. We touched on material properties as one type of important but elusive information. Occlusion also presents complex challenges. In contrast to rigid objects, a partial observation of a deformable object only partially constrains the positions of unobserved parts. Arnold et al. adopt a neural approach to estimate complete shape representations from partial observations, while quantifying remaining uncertainty in a granular localised fashion. Wang and Yamakawa estimate occluded areas in accordance with the object's material properties. Yet a different type of uncertainty is found in the modelling of object dynamics. When complex domain dynamics are learned from limited data, we cannot expect a model to be reliable for all inputs. Consequently, distinguishing what the model does and does not know becomes an important issue, as addressed by Arnold et al.

## Conclusion

We believe the work in this Research Topic presents exciting new developments on these and other challenges within DOM. The Research Topic co-editor team wishes to thank all authors for their contributions to this topic and to the field, and the reviewers for their efforts and insights.

## Author contributions

The author confirms being the sole contributor of this work and has approved it for publication.
